# Effects of Opioid Dependence on Visuospatial Memory and Its Associations With Depression and Anxiety

**DOI:** 10.3389/fpsyt.2019.00743

**Published:** 2019-10-23

**Authors:** Serenella Tolomeo, Fleur Davey, J. Douglas Steele, Alexander Mario Baldacchino

**Affiliations:** ^1^Department of Psychology, National University of Singapore, Singapore, Singapore; ^2^Centre for Family and Population Research, National University of Singapore, Singapore, Singapore; ^3^NHS Fife, Queen Margaret Hospital, Dunfermline, United Kingdom; ^4^School of Medicine, University of Dundee, Ninewells Hospital and Medical School, Dundee, United Kingdom; ^5^Division of Population and Behavioural Science, Medical School, University of St Andrews, St Andrews, United Kingdom

**Keywords:** memory, opioid dependence, heroin, methadone, buprenorphine, depression, anxiety

## Abstract

**Introduction:** The cognitive impact of opioid dependence is rarely measured systematically in everyday clinical practice even though both patients and clinicians accept that cognitive symptoms often occur in the opioid-dependent population. There are only a few publications which utilized computerized neuropsychological tests to assess possible impairments of visuospatial memory in opioid-dependent individuals either receiving opioid replacement therapy (ORT) or during subsequent short-term abstinence and the effects of anxiety and depression.

**Methods:** We assessed a cohort of 102 participants, comprising i) a stable opioid-dependent group receiving methadone maintenance treatment (MMT) (n = 22), ii) a stable opioid-dependent group receiving buprenorphine (BMT) (n = 20), iii) a current abstinent but previously opioid-dependent group (ABS) (n = 8), and iv) a control group who have never been dependent on opioids. The Cambridge Neuropsychological Automated Test Battery (CANTAB) neuropsychological tasks undertaken by participants included: Delayed Matching to Sample (DMS), Pattern Recognition Memory (PRM), Spatial Recognition Memory (SRM), and Paired Associate Learning (PAL) tasks. Three clinical measures were used to assess the severity of anxiety and depressive illness: Hospital Anxiety Scale-Hospital Anxiety Depression (HADA)-(HADD), Beck Depression Inventory (BDI), and Inventory of Depressive Symptomatology (self-report) (ISD-SR).

**Results:** The methadone- and buprenorphine-treated groups showed significant impairments (p < 0.001) in visuospatial memory tasks but not the abstinent group. Impairments in visuospatial memory strongly correlated with higher mood and anxiety symptom severity scores (p < 0.001).

**Discussion:** These results are broadly consistent with previous studies. Uniquely, though, here we report a strong relationship between visuospatial memory and depression and anxiety scores, which might suggest common illness mechanisms.

## Introduction

Substance misuse is a chronic condition often characterized by remissions and relapses (1). Individuals with a history of long-term opioid dependence may demonstrate cognitive impairments, primarily within the executive functioning domains (2–8).

These impairments have been linked to grey matter reductions in the prefrontal cortex, anterior mid-cingulate cortex, and basal ganglia (9), brain regions thought responsible for the regulation of cravings, pain, and emotional experience. In addition, other studies have reported how opioids affect memory, learning, and emotional disturbances (2, 3, 10, 11). Depression has long been associated with widespread cognitive deficits (12) which tend to worsen over a life span (13).

Specific memory tasks have shown to be sensitive and useful in detecting brain dysfunction in the temporal and amygdalo-hippocampal regions (14), which are consistently reported as functionally abnormal in mood disorders and sensation-seeking behaviors (15–17).

Importantly, these brain regions are also relevant to the neurobiology of substance misuse (18) with similar symptoms such as mood, anhedonia, and anxiety associated with drug dependence (19). These symptoms may represent a risk factor for the development of dependence and also may constitute a specific factor by which dependence is maintained, as well as strongly associated with major depressive disorder (MDD). However, depressive and anxiety symptoms have rarely been investigated in opioid dependence within a clinical environment.

Previous studies showed impairments in episodic memory (20), visual memory, verbal memory, information processing, problem solving (21), and spatial, tactile, and verbal memory (2) in heroin-, morphine-, and methadone-dependent participants. Curran and colleagues showed that a single dose of methadone could negatively impact on episodic memory in opiate users (20).

Previously, we have shown that visuospatial memory was impaired in chronic heroin and methadone-dependent participants, those maintained on methadone as part of opioid replacement therapy (ORT), or patients prescribed opioids for chronic pain (10). However, to our knowledge, there are no previous studies reporting the impact of opioid dependence on memory during short-term abstinence from opioids.

Here, we tested the following hypotheses:

- Visuospatial memory impairments are associated with current opioid exposure. Conversely, we therefore predicted that abstinence would be associated with no significant impairments.
- Cognitive impairments would correlate with mood and anxiety ratings. Specifically, we predicted that participants with higher depression and anxiety symptoms would have greater visuospatial memory impairments.

## Methods

Study approval was granted by the East of Scotland Research Ethics Committee (REC reference number: 06/S1401/32) and written informed consent obtained from all participants. National Health Service (NHS) Scotland Research Governance approval was provided by the NHS Fife Research and Development Department.

A total of 102 participants were *opportunistically* enrolled in this study with four groups: (i) a stable opioid-dependent group receiving methadone maintenance treatment (MMT) (n = 22), (ii) a stable opioid-dependent group receiving buprenorphine (BMT) (n = 20), (iii) a current abstinent but previously opioid-dependent group (ABS) (n = 8), and (iv) controls, with no history of illicit heroin, methadone, or buprenorphine use (n = 52). Patients had a diagnosis of *Diagnostic and Statistical Manual of Mental Disorders, Fourth Edition* (*DSM-IV*), Opioid Dependence and a history of poly-substance misuse with heroin as the primary “drug of choice” preceding initiation of MMT.

An extensive detailed screening was assessed by two clinicians (A.B. or F.D.), which included sociodemographic information collection and a semi-structured interview to obtain detailed previous histories of drug and alcohol use and current opioid dependence status (**Table 1** and **Supplementary Table 1**). Clinical histories and diagnoses were obtained using the structured Mini International Neuropsychiatric Interview (MINI Plus v 5.0) (22) together with a detailed review of individual clinical care records. The latter included recording the dose of methadone and buprenorphine that each participant received at the time of testing. A morphine equivalent calculation was performed in accordance to a previous publication by Vieweg et al. (23). Each methadone dose was multiplied by 20, and each buprenorphine dose was multiplied by 12 (23). Ongoing abstinence from illicit drug use was also objectively confirmed just prior to scanning with a urine drug test (24) using automated enzyme-mediated immunoassay to classify any detected drug (25). The Clinical Opioid Withdrawal Scale (COWS) was used to quantify the level of opioid withdrawal if present (26). Previous care records from Addiction Services, psychiatric notes, and general practitioners’ records confirmed the absence of hepatitis B and C and HIV. Other exclusion criteria included: past or current histories of psychotic disorders; post-traumatic stress disorder (PTSD); antisocial and borderline personality disorders; neurological and neurodevelopmental disorders; significant head injury; confirmed history of non-fatal overdose episodes; and co-occurring benzodiazepine, stimulant, and/or alcohol dependence.

**Table 1:** Demographic, clinical, and substance use history data.

	MMT (N = 22)	BMT (N = 20)	ABS (N = 8)	HC (N = 52)	Statistics
Number	22	20	8	51	
Age in years	33.6.	37.4	37.6	28.0	P < 0.001 MMT, BMT, ABS > HC***
NART	114.3 (5.2)	98.0 (13.5)	106.4 (15.6)	117.5 (6)	P < 0.001 BMT, ABS < HC***
HADA	6.0 (4.3)	4.8 (2.7)	4.0 (2.3)	3.5 (3.4)	P = 0.04
HADD	4.4 (3.5)	4.4 (2.9)	8.0 (1.5)	1.2 (2.3)	P < 0.001
BDI	12.4 (10)	9.9 (6.3)	9.0 (1.8)	3.7 (5.2)	P = 0.02
IDS-SR	17.8 (12)	12.6 (6.6)	14.0 (3.2)	7.9 (7.3)	P < 0.001
Fagerstrom (total score)	3.4 (2.3)	3.9 (2.3)	3.5 (2.8)		ns
OD (methadone or buprenorphine in mg)	73.4 (60.8)	11.0 (6.7)	–	–	P < 0.001 MMT > BMT***
Daily intake expressed as morphine equivalent dose in mg	1,835.5 (1,277)	888.0 (533)	–	–	P < 0.001 MMT > BMT***
Age when first used heroin in years	20.2 (4.4)	21.7 (5.4)	20.0 (4.7)	–	ns
Age when dependent on opioids in years	20.2 (4.4)	23.6 (5.9)	22.9 (8.5)	–	ns
Age when injecting opioids in years	21.8 (4.2)	24.8 (6)	22.7 (6.9)	–	ns
Years of opioid use	12.9 (4.4)	13.4 (6.7)	13.4 (7.6)	–	ns
Age when first used benzodiazepine in years	17.2 (5.8)	21.7 (7.7)	15.6 (6.6)		P < 0.04 MMT < BMT*
Days of benzodiazepine use in the last 30 days	–	–	–	–	–
Age when first used cocaine in years	17.3 (1)	21.9 (6.6)	18.3 (4.2)	–	ns
Days of cocaine use in last 30 days	–	–	–	–	–
Age when first used cannabis in years	13.3 (3.8)	15.8 (5.3)	13.1 (1.2)	–	ns
Days of cannabis use in last 30 days	–	–	–	–	–
Age when first used alcohol in years	10.5 (7.9)	15.1 (3)	13.0 (1.9)	–	0.04 MMT < BMT*
Days of alcohol use in last 30 days	–	–	–	–	–
Duration abstinence (days)	–	–	102.2 (61.3)	–	–

Values are mean (SD); MMT, methadone maintenance treatment group; BMT, buprenorphine maintenance treatment group; ABS, abstinent group; HC, healthy control group; N, total number; HADA, Hospital Anxiety Scale; HADD, Hospital Anxiety Depression; BDI, Beck Depression Inventory; IDS-SR, Inventory of Depressive Symptomatology (self-report); NART, National Adult Reading Test; significance * = p = 0.05, *** = p < 0.001; ns, non-significant; mg, milligrams; OD, opioid dose (methadone or buprenorphine).

Current and premorbid intelligence was estimated using the Wechsler Abbreviated Scale of Intelligence (WASI) and National Adult Reading Test (NART) (27, 28).

### Visuospatial Memory Tasks

The Cambridge Neuropsychological Automated Test Battery (CANTAB, www.camcog.com) comprises a series of computerized memory tasks (29). As previously reported, the following tasks have shown specificity to detect impairments in *visual memory performance* [Delayed Matching to Sample (DMS), Pattern Recognition Memory (PRM), Spatial Recognition Memory (SRM), and Paired Associate Learning (PAL)] and *spatial memory performance* [Spatial Span Task (SSP) and Spatial Working Memory (SWM)] (10).

### Depression and Anxiety Rating Scales

Three clinical measures were used to assess the severity of anxiety and depressive illness: the Hospital Anxiety and Depression Scale (HADS) (30), Beck Depression Inventory (BDI) (23), and Inventory of Depressive Symptomatology (IDS): clinician (IDS-C) and self-report (IDS-SR) (31).

HADS is commonly used to determine depression and anxiety. It is a 14-item scale with 7 items that relate to depression (HADD) and 7 items to anxiety (HADA) (30). BDI and IDS are self-report inventories, and they have been mostly used to assess depression and anhedonia (32, 33). BDI demonstrated high internal consistency, with an alpha coefficient of 0.82 (34). Similarly, IDS demonstrated strong internal consistency, with an alpha coefficient of 0.88 (35).

### Statistical Analysis

Data meeting assumptions of normality and homogeneity of variance were analyzed using analysis of variance (ANOVA) (36). All other data were compared using Mann–Whitney test. Preliminary analysis of all the experimental and control groups separately indicated that the samples did not come from normally distributed populations with the same standard deviation. We used a *post hoc Bonferroni* correction in order to control for family-wise error for unplanned tests. Mann–Whitney U tests established that NART, age, and smoking history needed to be used as covariates for hypothesis testing.

A general linear model was performed with “groups” as a factor and “visuospatial memory task performances” as dependent variables using analysis of covariance (ANCOVA). To explore the potential contribution of the impact of depression and anxiety scores on memory task performance, we added an additional correlational analysis within the ANCOVA.

Data were analyzed using the Statistical Package for the Social Science (SPSS) version 24 (SPSS Inc.) in Windows 10 on a PC computer. P values < 0.05 were considered significant.

## Results

### Demographic Characteristics

Demographic and clinical characteristics are presented in **Table 1**. Participants and controls were matched on the basis of gender (all males). The MMT, BMT, and ABS groups were older than the healthy controls (HCs) (p < 0.001). The HC group had higher estimated premorbid IQ (p < 0.001) according to the NART than the BMT and ABS groups. The mean morphine equivalent daily dose for the MMT group was significantly higher than the BMT (p < 0.001). Urine analyses confirmed complete absence of recent heroin, amphetamine, benzodiazepine, and cocaine prior to neuropsychological testing. The MMT group reported they first drank alcohol and consumed benzodiazepine approximately 4.5 years prior to the BMT cohort (p < 0.04). There were no significant group differences identified on several clinical substance history data such as: age when they first used heroin (p = 0.6), age when dependent on heroin (p = 0.2), or age when injecting opioids (p = 0.3). The MMT, BMT, and ABS were well matched with regard to age when they first used cocaine (p = 0.15) and cannabis (p = 0.13).

### Visual Memory

#### Performance on DMS

There was a significant effect of group on the percentage of correct responses for DMS [F(4, 78) = 7.5, p < 0.001]. *Post hoc* Bonferroni comparisons showed that participants from the MMT and BMT groups made significantly more errors than the ABS and HC groups (p = 0.03 and p < 0.001, respectively). There was a significant effect of group on the percentage of correct responses for DMS [F(4, 78) = 7.4, p < 0.001]. *Post hoc* Bonferroni comparisons showed that participants from the MMT and BMT groups made significantly more errors than the ABS and HC groups (p = 0.02 and p < = .001, respectively).

**Figure 1:**
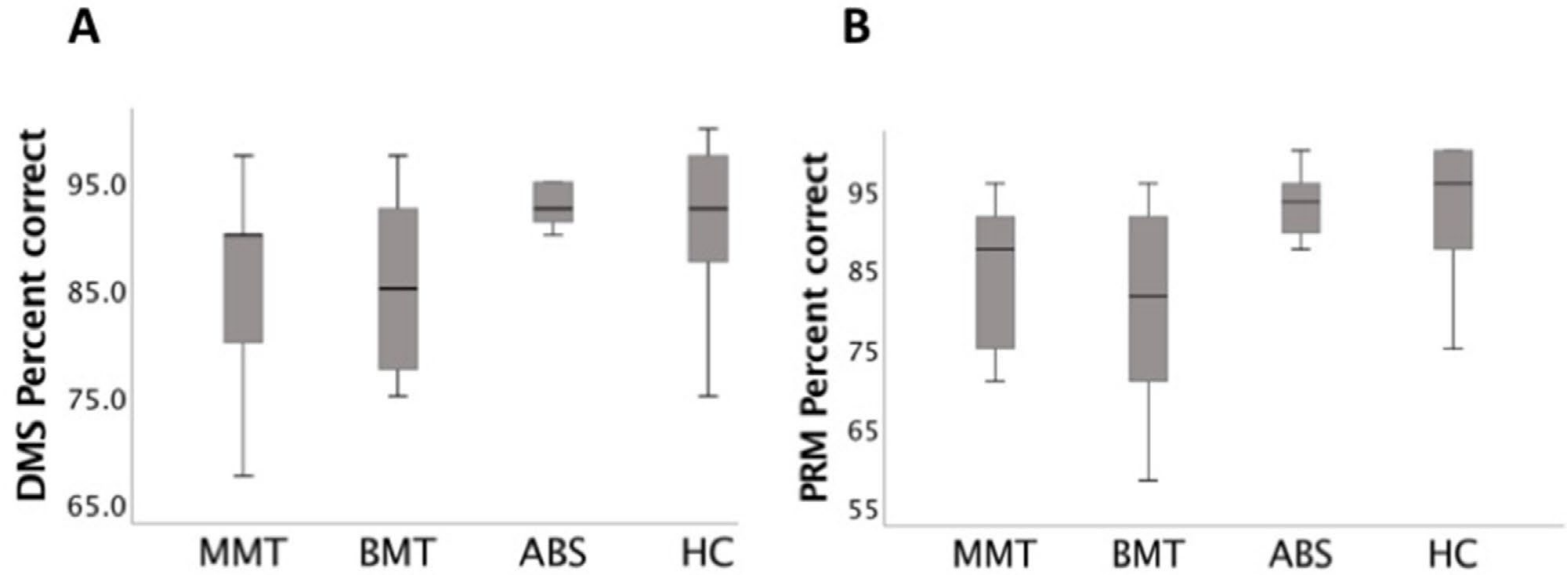
**(A)** Delayed Matching to Sample (DMS) task (% correct) box plots: the stable opioid-dependent group receiving methadone maintenance treatment (MMT) and that receiving buprenorphine (BMT) made significantly more errors than the abstinent but previously opioid-dependent group (ABS) and healthy controls (HCs) (p < 0.001) groups. **(B)** Pattern Recognition Memory (PRM) task (% correct) box plots: the MMT and BMT made significantly more errors than the ABS and HC (p < 0.001) groups.

More details are reported in **Table 2** and **Figure 1**.

**Table 2:** Summary of neuropsychological findings for visual and spatial memory.

Memory and learning measures	MMT (N = 22)	BMT (N = 20)	ABS (N = 8)	HC (N = 52)	Statistics
Visual Memory					
DMS % correct	84.5 (11.6)	80.0 (15)	92.8 (2.1)	92.5 (5.9)	P < 0.001, MMT, BMT < ABS, HC***
DMS % correct (all delays)	80.2 (14.8)	75.6 (18.5)	91.6 (3.9)	90.7 (7.6)	P < 0.001, MMT, BMT < ABS, HC***
PRM % correct	83.8 (10.1)	80.1 (11.7)	90.2(0.09)	93.2 (4.3)	P < 0.001, MMT, BMT < ABS, HC***
SRM mean correct latency	1,997 (377)	2,743 (1,138)	2,150 (454)	1,882 (555)	P = 0.001, BMT > HC***
PAL total errors adjusted	125.7 (101)	29.9 (34.6)	11.0 (9)	57.0 (90)	P = 0.001, MMT, BMT > ABS, HC***
PAL first trial memory score	8.5 (0.8)	17.9 (4.5)	19.7 (3)	16.4 (9)	P = 0.001, MMT < HC, ABS***
Spatial Memory					
SWM between errors	8.8 (15.9)	33.4 (21.4)	22.7 (16.2)	16.6 (21.9)	P = 0.003, BMT > MMT, HC***
SWM strategy	13.1 (14.9)	32.9 (6.9)	31.7 (6)	21.3 (13.4)	P < 0.001, MMT < BMT, ABS***

Values are mean (SD); significance *** = P < 0.001; DMS, Delayed Matching to Sample; PRM, Pattern Recognition Memory, SRM, Spatial Recognition Memory; PAL, Paired Associate Learning; SWM, Spatial Working Memory; N, total number.

#### Performance on PRM, SRM, and PAL

**Figure 2:**
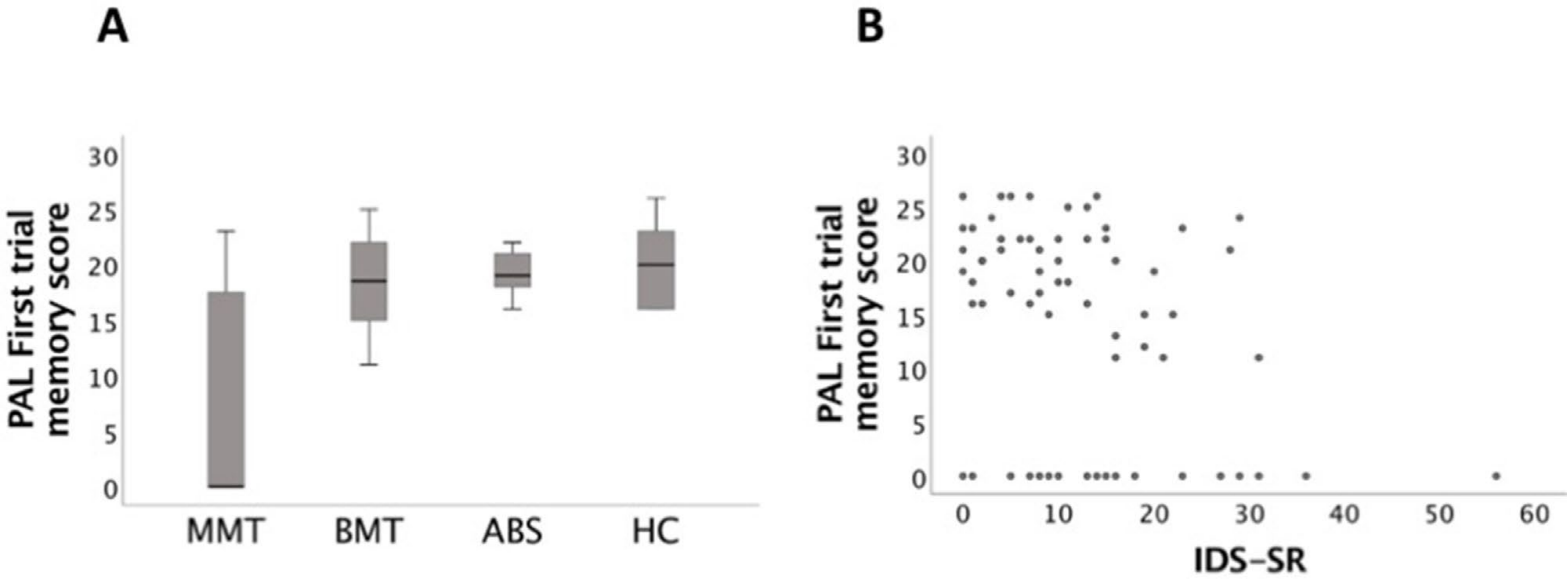
**(A)** Paired Associate Learning (PAL) (first trial memory score) box plots: those in the MMT group were significant more impaired than the ABS and HC groups (p < 0.001). **(B)** PAL (first trial memory score) task significantly correlated with Inventory of Depressive Symptomatology (self-report) (IDS-SR) (p = 0.005).

There was a significant effect of group on the percentage of correct responses for the PRM task [F(4, 60) = 9.3, p < 0.001] and on the mean correct latency for the SRM task [F(4, 60) = 6.4, p < 0.001]. Similarly, there was a significant effect of group on the total adjusted errors on the PAL task [F(4, 75) = 6.1, p < 0.001] and on PAL first trial memory [F(4, 75) = 5.7, p < 0.001] (see **Figure 2**).

### Spatial Memory

#### Performance on SSP and SWM

There was a significant effect of group on the SSP task (span length) [F(4, 75) = 10.5, p < 0.001]. The BMT and ABS groups (a) made significantly more errors (between errors) [F(4, 75) = 5, p < 0.003] and (b) presented with a poorer strategy on the SWM task [F(4, 75) = 9.8, p < 0.001].

### Depression and Anxiety and Visuospatial Memory Performance

Higher HADA anxiety, BDI, and IDS-SR depression scores were significantly correlated with PAL (total error adjusted [r (66) = 0.3, p = 0.01, r (66) = 0.25, p = 0.04, r (64) = 0.3, p < 0.005, respectively]). Similarly, higher HADA, BDI, and IDS-SR scores were significantly associated with PAL (first trial memory score) [r (66) = 0.3, p = 0.007, r (66) = 0.28, p = 0.02, r (64) = 0.4, p = 0.001, respectively]. DMS (% correct) significantly correlated with BDI [r (66) = 0.3, p = 0.01] (see **Table 3**).

**Table 3:** Correlations between depression and anxiety and visuospatial performance.

	HADA	BDI	IDS-SR
PAL (total error adjusted)	0.3**	0.25*	0.3**
PAL (first trial memory score)	0.3**	0.28*	0.4***
DMS (% correct)	–	0.3**	–

* indicates p < 0.05, ** indicates p < 0.01, *** indicates p < 0.001.

## Discussion

In this clinically well-characterized study, we have demonstrated that memory for visually presented patterns and spatial locations was impaired in individuals on ORT. This is consistent with previous studies utilizing computerized CANTAB assessment with individuals on ORT and HCs. These studies revealed that individuals on ORT exhibited impairments in comparison to controls on the PRM task (2, 3) and on the PAL task (21). In a recent meta-analysis by Baldacchino and colleagues (37), short-term memory impairments were not present in the abstinence cohorts. This is consistent with our present results, as cognitive impairments were not present in the abstinent group for both visual and spatial memory tasks.

We previously reported that cognitive processes particularly associated with the prefrontal cortex are disrupted during chronic opioid use but not during abstinence (9). Our results could be explained by frontal lobe dysfunction (9, 38–40), which can potentially cause impairments on tasks requiring optimal memory function with patients receiving ORT. In addition, the identified impairments within the opioid-dependent groups on ORT point to specific correlations with depression and anxiety, particularly with tasks sensitive to the anatomical location of the medial temporal lobe.

This is consistent with numerous studies in healthy volunteers identifying the medial temporal lobe, such as the hippocampus and amygdala, as the area where memory-sensitive tasks are encoded (41, 42). Of specific interest, the medial temporal lobe regions have been reported 1) as structurally abnormal in depressive disorder (16) and 2) as one of the main putative candidate regions for both the development and the maintenance of dependence (18) and depression (43).

Regarding possible limitations of the present study, we recruited only males, so these findings shouldn’t be generalized to females (44). Drug use and clinical histories were collected based upon self-report, and no blood, hair, or saliva samples were available to confirm the accuracy of the information given; however, our study did acquire urine drug screen analysis to confirm the absence of recent illicit drug use prior to every session. Additionally, the present study recruited well-matched subjects with regard to their previous drug history in the experimental groups and excluded regular and dependent users of most psychoactive substances, such as alcohol and benzodiazepines, as they have been shown to profoundly impact neuropsychological performance (18). We couldn’t control the effect of nicotine, which may have influenced our results due to its known neuropsychological effects on visual and spatial memory (45). The burprenorphine group had a significant lower morphine equivalent dose than the methadone group, which may impact our findings; however, no statistically significant correlations were present. Larger studies with long-term abstinence are required to fully validate the observed reversibility and possible extinction of these impairments.

### Clinical Relevance

Patients’ questions about the effects of opioid dependence on memory and its impact during abstinence cannot comprehensively be answered, due to a current lack of research in this area (10). More data are required on the consequences of opioid dependence on memory in order to evaluate the acceptability of differential treatments, such as methadone and buprenorphine, and perhaps maximize abstinence periods (46). Previous studies have indicated the importance of detecting memory impairments using highly structured and extensive neuropsychological batteries. This is further highlighted in the present study, indicating that opioid-dependent individuals have memory loss in both visual and spatial domains. Early identification of memory impairments associated with opioid dependence could improve the current standard clinical method of assessment. Elucidating the cognitive and neural mechanisms responsible for the formation and maintenance of opioid-related associative dependence has the potential for opening up new therapeutic trajectories during both the prevention and/or reversal of the significant effects on memory and learning, which may be a vulnerability for development and maintenance of opioid dependence. Notably, our results highlight the possibility that opioid-dependent individuals may benefit from focused treatments for depression and anxiety symptoms during ORT.

In particular, understanding the underlying neurocognitive and brain substrates linked to a dual close relationship between comorbid substance misuse and mood states may (a) reveal potential new interventions for the treatment of protracted opioid dependence and/or relapse (18) and (b) provide the required biomarkers to create predictive algorithms to detect early dependence and abstinence (6, 7).

## Conclusion

In summary, our results found that opioid-dependent participants exhibited visuospatial memory impairments closely associated with depression and anxiety scores. These impairments were not present in short-term abstinence, suggesting reversible impairments. Further studies need to explore the effect that mood plays in cognitive impairments observed in this and other dependent populations (e.g. nicotine and alcohol). Indeed, identifying and characterizing the visuospatial memory abilities and their potential mechanisms of action may be of crucial importance in identifying potential common mechanisms controlling the switch from the non-dependent to substance-dependent states and ultimately achieving abstinence in the opioid-dependent population.

## Data Availability Statement

The datasets generated for this study are available on request to the corresponding author.

## Ethics Statement

Study approval was granted by the East of Scotland Research Ethics Committee (REC reference number: 06/S1401/32) and written informed consent obtained from all participants. National Health Service (NHS) Scotland Research Governance approval was provided by the NHS Fife Research and Development Department.

## Author Contributions

ST wrote the first draft of the manuscript with AB’s input and created the figures and tables. FD and JS provided revisions to versions of the draft manuscript. ST formatted the manuscript for publication.

## Funding

This study was partly funded by an unrestricted educational grant provided by Schering-Plough, the Anonymous Trust, and Indivior. The funding sources had no role in the design or conduct of the study and interpretation of the data.

## Conflict of Interest

ST has received unrestricted research funding from Indivior, Lundbeck Foundation, and Merck Serono. JS has received research funding from Medical Research Council (MRC) and Wellcome Trust. He has received research funding *via* an honorarium associated with a lecture from Wyeth and unrestricted educational grants from Indivior and Schering Plough. AB has received research project funding from MRC, Chief Scientist Office (CSO), Schering-Plough, Merck Serono, Lundbeck, and Indivior. FD declares that the research was conducted in the absence of any commercial or financial relationships that could be construed as a potential conflict of interest.

## References

[1] Wolf JP Ponicki WR Kepple NJ Gaidus A Are community level prescription opioid overdoses associated with child harm? A spatial analysis of California zip codes, 2001–2011. Drug Alcohol Depend (2016) 166:202–8. 10.1016/j.drugalcdep.2016.07.014 PMC4987103 27496625

[2] Ornstein TJ Iddon JL Baldacchino AM Sahakian BJ London M Everitt BJ Profiles of cognitive dysfunction in chronic amphetamine and heroin abusers. Neuropsychopharmacology (2000) 23(2):113. 10.1016/S0893-133X(00)00097-X 10882838

[3] Ersche KD Clark L London M Robbins TW Sahakian BJ Profile of executive and memory function associated with amphetamine and opiate dependence. Neuropsychopharmacology (2006) 31(5):1036. 10.1038/sj.npp.1300889 16160707 PMC1867318

[4] Ersche KD Sahakian BJ The neuropsychology of amphetamine and opiate dependence: implications for treatment. Neuropsychol Rev (2007) 17(3):317–36. 10.1007/s11065-007-9033-y PMC3639428 17690986

[5] Baldacchino A Balfour DJK Matthews K Impulsivity and opioid drugs: differential effects of heroin, methadone and prescribed analgesic medication. Psychol Med (2015) 45(6):1167–79. 10.1017/S0033291714002189 25171718

[6] Verdejo-García A Pérez-García M Profile of executive deficits in cocaine and heroin polysubstance users: common and differential effects on separate executive components. Psychopharmacology (2007) 190(4):517–30. 10.1007/s00213-006-0632-8 17136401

[7] Tolomeo S Gray S Matthews K Steele JD Baldacchino A Multifaceted impairments in impulsivity and brain structural abnormalities in opioid dependence and abstinence. Psychol Med (2016a) 46(13):2841–53. 10.1017/S0033291716001513 27452238

[8] Tolomeo S Matthews K Steele D Baldacchino A Compulsivity in opioid dependence. Prog Neuropsychopharmacol Biol Psychiatry (2018) 81:333–9. 10.1016/j.pnpbp.2017.09.007 28918267

[9] Tolomeo S Christmas D Jentzsch I Johnston B Sprengelmeyer R Matthews K A causal role for the anterior mid-cingulate cortex in negative affect and cognitive control. Brain (2016b) 139(6):1844–54. 10.1093/brain/aww069 27190027

[10] Baldacchino A Tolomeo S Balfour DJ Matthews K Profiles of visuospatial memory dysfunction in opioid-exposed and dependent populations. Psychol Med (2018) 1–11. 10.1017/S0033291718003318 30457069

[11] Kutlu MG Gould TJ Effects of drugs of abuse on hippocampal plasticity and hippocampus-dependent learning and memory: contributions to development and maintenance of addiction. Learn Mem (2016) 23(10):515–33. 10.1101/lm.042192.116 PMC5026208 27634143

[12] Austin MP Mitchell P Goodwin GM Cognitive deficits in depression: possible implications for functional neuropathology. Br J Psychiatry Suppl (2001) 178(3):200–6. 10.1192/bjp.178.3.200 11230029

[13] Albert KM Potter GG McQuoid DR Taylor WD Cognitive performance in antidepressant-free recurrent major depressive disorder. Depression and Anxiety (2018) 35(8):694–9. 10.1002/da.22747 PMC6105441 29637661

[14] Owen AM Sahakian BJ Semple J Polkey CE Robbins TW Visuo-spatial short-term recognition memory and learning after temporal lobe excisions, frontal lobe excisions or amygdalo-hippocampectomy in man. Neuropsychologia (1995) 33(1):1–24. 10.1016/0028-3932(94)00098-A 7731533

[15] Johnston BA Tolomeo S Gradin V Christmas D Matthews K Douglas Steele J Failure of hippocampal deactivation during loss events in treatment-resistant depression. Brain (2016) 138(9):2766–76. 10.1093/brain/awv177 26133661

[16] Schmaal L Veltman DJ van Erp TG Sämann PG Frodl T Jahanshad N Subcortical brain alterations in major depressive disorder: findings from the ENIGMA major depressive disorder working group. Mol Psychiatry (2016) 21(6):806 10.1038/mp.2015.69 26122586 PMC4879183

[17] Fornaro M Ventriglio A De Pasquale C Pistorio ML De Berardis D Cattaneo CI Sensation seeking in major depressive patients: relationship to sub-threshold bipolarity and cyclothymic temperament. J Affect Dis (2013) 148(2–3):375–83. 10.1016/j.jad.2013.01.002 23414573

[18] Koob GF Volkow ND Neurocircuitry of addiction. Neuropsychopharmacology (2010) 35(1):217. 10.1038/npp.2009.110 19710631 PMC2805560

[19] Koob GF The dark side of emotion: the addiction perspective. Eur J Pharmacol (2015) 753:73–87. 10.1016/j.ejphar.2014.11.044 25583178 PMC4380644

[20] Curran HV Kleckham J Bearn J Strang J Wanigaratne S Effects of methadone on cognition, mood and craving in detoxifying opiate addicts: a dose-response study. Psychopharmacology (2001) 154(2):153–60. 10.1007/s002130000628 11314677

[21] Darke S Sims J McDonald S Wickes W Cognitive impairment among methadone maintenance patients. Addiction (2000) 95(5):687–95. 10.1046/j.1360-0443.2000.9556874.x 10885043

[22] Sheehan DV Lecrubier Y Sheehan KH Amorim P Janavs J Weiller E The Mini-International Neuropsychiatric Interview (MINI): the development and validation of a structured diagnostic psychiatric interview for DSM-IV and ICD-10. J Clin Psychiatry (1998) 59:22–23. 10.1037/t18597-000 9881538

[23] Vieweg WVR Lipps WFC Fernandez A Opioids and methadone equivalents for clinicians. Prim Care Companion J Clin Psychiatry (2005) 7(3):86. 10.4088/PCC.v07n0301 16027761 PMC1163279

[24] Armbruster DA Krolak JM Screening for drugs of abuse with the Roche ONTRAK assays. J Anal Toxicol (1992) 16(3):172–5. 10.1093/jat/16.3.172 1522711

[25] Wilson JF Smith BL Toseland PA Williams J Burnett D Hirst AD External quality assessment of techniques for the detection of drugs of abuse in urine. Annal Clin Biochem (1994) 31(4):335–42. 10.1177/000456329403100405 7979098

[26] Wesson DR Ling W The Clinical Opiate Withdrawal Scale (COWS). J Psychoactive Drugs (2003) 35(2):253–9. 10.1080/02791072.2003.10400007 12924748

[27] Nelson HE Willison J National Adult Reading Test (NART). Windsor: Nfer-Nelson (1991).

[28] Woerner C Overstreet K Wechsler Abbreviated Scale of Intelligence (WASI). San Antonio, TX: The Psychological Corporation (1999).

[29] Robbins TW James M Owen AM Sahakian BJ Lawrence AD McInnes L A study of performance on tests from the CANTAB battery sensitive to frontal lobe dysfunction in a large sample of normal volunteers: implications for theories of executive functioning and cognitive aging. J Int Neuropsychol Soc (1998) 4(5):474–90. 10.1017/S1355617798455073 9745237

[30] Zigmond AS Snaith RP The hospital anxiety and depression scale. Acta Psychiatr Scand (1983) 67(6):361–70. 10.1111/j.1600-0447.1983.tb09716.x 6880820

[31] Beck AT Ward C Mendelson M Mock J Erbaugh J Beck Depression Inventory (BDI). Arch Gen Psychiatry (1961) 4(6):561–71. 10.1001/archpsyc.1961.01710120031004 13688369

[32] Rush AJ Carmody T Reimitz PE The Inventory of Depressive Symptomatology (IDS): clinician (IDS-C) and self-report (IDS-SR) ratings of depressive symptoms. Int J Methods Psychiatr Res (2000) 9(2):45–59. 10.1002/mpr.79

[33] Deakin JFW The origins of ‘5-HT and mechanisms of defence’ by Deakin and Graeff: a personal perspective. J Psychopharmacol (2013) 27(12):1084–9. 10.1177/0269881113503508 24067790

[34] Beck AT Steer RA Carbin MG Psychometric properties of the Beck Depression Inventory: Twenty-five years of evaluation. Clin Psychol Rev (1988) 8(1):77–100.

[35] Rush AJ Giles DE Schlesser MA Fulton CL Weissenburger J Burns C The inventory for depressive symptomatology (IDS): preliminary findings. Psychiatry Res (1986) 18(1):65–87. 3737788 10.1016/0165-1781(86)90060-0

[36] Winer BJ Brown DR Michels KM Statistical principles in experimental design. 3rd ed New York: McGraw Hill (1991).

[37] Baldacchino A Armanyous M Balfour DJ Humphris G Matthews K Neuropsychological functioning and chronic methadone use: a systematic review and meta-analysis. Neuroscience & Biobehavioral Reviews (2017) 73:23–38. 27913280 10.1016/j.neubiorev.2016.11.008

[38] Lyoo IK Pollack MH Silveri MM Ahn KH Diaz CI Hwang J Prefrontal and temporal gray matter density decreases in opiate dependence. Psychopharmacology (2006) 184(2):139–44. 10.1007/s00213-005-0198-x 16369836

[39] Liu H Hao Y Kaneko Y Ouyang X Zhang Y Xu L Frontal and cingulate gray matter volume reduction in heroin dependence: Optimized voxel‐based morphometry. Psychiatry Clin Neurosci (2009) 63(4):563–8. 10.1111/j.1440-1819.2009.01989.x 19531112

[40] Yuan Y Zhu Z Shi J Zou Z Yuan F Liu Y Lee TM Weng X Gray matter density negatively correlates with duration of heroin use in young lifetime heroin-dependent individuals. Brain and Cognition (2009) 71(3):223–8. 10.1016/j.bandc.2009.08.014 19775795

[41] Maguire EA Frackowiak RS Frith CD Learning to find your way: a role for the human hippocampal formation. Proc R Soc Lond B Biol Sci (1996) 263(1377):1745–50. 10.1098/rspb.1996.0255 9025317

[42] Owen AM Morris RG Sahakian BJ Polkey CE Robbins TW Double dissociations of memory and executive functions in working memory tasks following frontal lobe excisions, temporal lobe excisions or amygdalo-hippocampectomy in man. Brain (1996) 119(5):1597–615. 10.1093/brain/119.5.1597 8931583

[43] Hill D Garner D Baldacchino AM Comparing neurocognitive function in individuals receiving chronic methadone or buprenorphine for the treatment of opioid dependence: a systematic review. Heroin Addict Relat Clin Probl (2018) 20(5):35–49.

[44] Ardila A Rosselli M Matute E Inozemtseva O Gender differences in cognitive development. Develop Psychol (2011) 47(4):984. 10.1037/a0023819 21744957

[45] Conti AA McLean L Tolomeo S Steele JD Baldacchino A Chronic tobacco smoking and neuropsychological impairments: a systematic review and meta-analysis. Neurosci Biobehav Rev (2018). 10.1016/j.neubiorev.2018.11.017 30502351

[46] Verdejo-García AJ Perales JC Pérez-García M Cognitive impulsivity in cocaine and heroin polysubstance abusers. Addict Behav (2007) 32(5):950–66. 10.1016/j.addbeh.2006.06.032 16876962

