# Supplementary material for: Effects of Opioid Dependence on Visuospatial Memory and Its Associations With Depression and Anxiety
Source: Front Psychiatry. 2019 Oct 23;10:743. doi: 10.3389/fpsyt.2019.00743 (PMC6820290; doi:10.3389/fpsyt.2019.00743)
Supplement: Supplementary file 1 [file Table_1.docx]

**Supplementary Table 1: Summary of clinical screening and diagnostic instruments and cognitive tests and visuospatial outcome measures**

| **Screening Tests** | **Measures** |
| --- | --- |
| Mini International Neuropsychiatric Instrument (MINI) *v5.0.* | Diagnosis of 15 Axis 1 and 1 Axis 2 DSM-4 psychiatric illnesses and substance misuse/dependence presented in 24 modules. |
| Case records from addiction, psychiatric and General Practitioner’s Services. | Identification of non-fatal overdose episodes  Confirming a history or not of epilepsy and other neurological phenomenon including learning disabilities.  Confirming a diagnosis of Hepatitis B, C and HIV  Validating medical and psychiatric histories  Validating substance misuse career and current drug and alcohol use.  Scottish Index of Multiple Deprivation (SIMD). |
| Post Trauma Amnesia Questionnaire *v1.0.* | Information on head and other cerebral insults and consequential post trauma amnesia. |
| On site and urine analysis for illicit drug screen. | Using on site drug test kits for presence of amphetamine, cannabinoids, opioids, benzodiazepine and cocaine. Subsequent lab investigations used ELISA followed up by TLC methodologies. |
| **Diagnostic Tests** | **Measures** |
| Mini International Neuropsychiatric Instrument (MINI) (Section L) *v5.0.* | Diagnosis of opioid misuse/dependence (lifetime and current). |
| Maudsley Addiction Profile (MAP) *v1.0.* | Information on substance misuse history, health risk behaviour, physical and psychological health, personal and social functioning in the last 30 days. |
| Fagerström Test for Nicotine Dependence (FTND) *v1.0.* | 6 items with a diagnostic score ranging from 0 (no nicotine dependence) to 10 (very severe nicotine dependence). |
| Clinical Opiate Withdrawal Scale (COWS) *v1.0.* | 11 objective and subjective symptoms of opioid withdrawal. Scores ranged from 0-48. |
| On site and urine analysis for illicit drug screen. | Using on site drug test kit for presence of amphetamine, cannabinoids, opioids, benzodiazepine and cocaine. Subsequent lab investigations used ELISA followed up by TLC methodologies. |
| **Cognitive Tests** | **Measures** |
| National Adult Reading Test (NART) *v2.0.* | WAIS-R Full Scale (IQ=70-131), Verbal Scale (IQ=72-127) and Performance Scale (IQ=74-128). |
| **CANTAB*eclipse v3.0.*** |  |
| (a) **Visual Memory** |  |
| Delayed Matching to Sample (DMS). | Speed of response (latency)  Mean total correct trials (0ms, 4ms, 12ms)*. |
| Pattern Recognition Memory (PRM). | Total Number of Correct Trials* and Mean Correct Latency. |
| Spatial Recognition Memory (SRM). | Total number of Correct Trials* and Mean Correct Latency. |
| Paired Associate Learning (PAL). | Mean Total Number of Errors*, Memory Score*.  Stages Completed and on First Trial. |
| (b) **Spatial Memory** |  |
| Spatial Span (SSP). | Span Length and Total Errors*, Mean Time to First and Last Response. |
| Spatial Working Memory (SWM). | Total Search Errors (mean, 4, 6, 8 moves problem)*.  Between, Within and Double Search Errors (mean, 4, 6, 8 move problems)*.  Strategy Score |
| (c) **Impulsivity** |  |
| Cambridge Gambling Task (CGT) | Risk Adjustment, Delay Aversion |

*= Outcomes measuring visuospatial memory.
